# Establishment and characterization of the immortalized porcine lung-derived mononuclear phagocyte cell line

**DOI:** 10.3389/fvets.2022.1058124

**Published:** 2022-11-18

**Authors:** Takato Takenouchi, Kentaro Masujin, Shunichi Suzuki, Seiki Haraguchi, Kanae Hiramatsu, Takehiro Kokuho, Hirohide Uenishi

**Affiliations:** ^1^Institute of Agrobiological Sciences, National Agriculture and Food Research Organization, Tsukuba, Japan; ^2^Division of Transboundary Animal Disease Research, National Institute of Animal Health, National Agriculture and Food Research Organization, Tokyo, Japan; ^3^Division of Infectious Animal Disease Research, National Institute of Animal Health, National Agriculture and Food Research Organization, Tsukuba, Japan

**Keywords:** African swine fever virus, immortalized PLuM (IPLuM), macrophages, mononuclear phagocytes (MNP), porcine lung-derived MNP (PLuM)

## Abstract

Mononuclear phagocytes (MNP), including monocytes, dendritic cells (DC), and macrophages, play critical roles in innate immunity. MNP are abundant in the lungs and contribute to host defense against airborne agents and pulmonary immune homeostasis. In this study, we isolated porcine lung-derived MNP (PLuM) from primary cultures of parenchymal lung cells and then immortalized them by transferring the SV40 large T antigen gene and porcine telomerase reverse transcriptase gene using lentiviral vectors. The established cell line, immortalized PLuM (IPLuM), expressed DC/macrophage markers; i.e., CD163, CD172a, and major histocompatibility complex class II, whereas they did not express a porcine monocyte-specific marker, CD52. The expression patterns of these cell surface markers indicate that IPLuM originate from the DC/macrophage lineage rather than the monocyte lineage. The bacterial cell wall components muramyl dipeptide and lipopolysaccharide induced the production of the interleukin-1 family of pro-inflammatory cytokines in IPLuM. Phagocytotic activity was also detected by time-lapse fluorescence imaging of live cells when IPLuM were cultured in the presence of pHrodo dye-conjugated *E. coli* BioParticles. It is worth noting that IPLuM are susceptible to African swine fever virus infection and support the virus' efficient replication *in vitro*. Taken together, the IPLuM cell line may be a useful model for investigating host-agent interactions in the respiratory microenvironments of the porcine lung.

## Introduction

The mononuclear phagocyte system is an important part of the innate immune system (1). Mononuclear phagocytes (MNP) comprise monocytes, dendritic cells (DC), and macrophages and are characterized by their phagocytosis and antigen presentation abilities (1). DC are professional antigen-presenting cells, which initiate adaptive immune responses (1). Macrophages are professional phagocytes and are highly specialized for the removal of dead cells and cellular debris (1). Although monocytes were historically considered to be a precursor of DC/macrophages, recent evidence has demonstrated the distinct origins of DC, macrophages, and monocytes (2). MNP play especially critical roles in tissues that are in direct contact with the outside world, such as the intestine, skin, and lungs (3).

Regarding MNP of the porcine lung, the DC/macrophages can be segregated into at least six subpopulations; i.e., conventional DC1 (cDC1), cDC2, monocyte-derived DC, monocyte-derived intravascular macrophages, interstitial macrophages, and alveolar macrophages (4). Among them, *in vitro* cultures of porcine primary alveolar macrophages (PAM) have been frequently used for the identification and characterization of porcine viral pathogens, such as porcine reproductive and respiratory syndrome virus (PRRSV) and African swine fever virus (ASFV) (5, 6). PAM are selectively collected during bronchoalveolar lavage procedures, while all subpopulations of DC/macrophages can be recovered from primary cultures of porcine parenchymal lung cells (5).

In this study, we collected porcine lung-derived MNP (PLuM) from mixed primary cultures of porcine parenchymal lung cells, as described in our previous study (7). We further established a novel immortalized PLuM (IPLuM) cell line and analyzed the cells' phenotypic characteristics and susceptibility to infection by ASFV.

## Materials and methods

### Ethics statement

The protocols for the use of animals were approved by the animal care committee of the Institute of Agrobiological Sciences (#H28-P04) and the National Institute of Animal Health (NIAH) (#20-046), National Agriculture and Food Research Organization.

### Isolation of PLuM

The lung parenchyma was dissected out from a 1-month-old crossbred pig and cut into small pieces with scissors, and the tissue pieces were digested by incubating them with collagenase-dispase (Roche Diagnostics, Basel, Switzerland)/Dulbecco's phosphate-buffered saline (DPBS) solution (1 mg/mL) containing DNase I (Roche Diagnostics; 40 μg/mL) for 1 h at 37°C. Then, the digested tissue fragments were collected and re-suspended in growth medium composed of Dulbecco's modified Eagle's medium (DMEM) (Sigma, St. Louis, MO) containing 10% heat-inactivated fetal bovine serum (FUJIFILM Wako Pure Chemical Corp., Osaka, Japan) and supplemented with 25 μM monothioglycerol (FUJIFILM Wako), 10 μg/mL insulin (Sigma), streptomycin-penicillin (100 μg/mL and 100 U/mL, respectively) (Nacalai Tesque, Inc., Kyoto, Japan), and 5 μg/mL Fungin (InvivoGen, San Diego, CA). The tissue suspension was added to T-75 tissue culture flasks (Sumitomo Bakelite Co., Ltd., Tokyo, Japan) and cultured at 37°C in a humidified atmosphere of 95% air/5% CO_2_. The culture medium was replaced every 3–4 days. After 1–2 weeks, a sheet-like cell monolayer formed, and spherical cells containing PLuM appeared on the cell sheet. The cells loosely attached to the cell sheet and so were harvested from the culture supernatant by centrifugation (1500 rpm for 5 min). Since PLuM readily attach to non-tissue culture-grade petri dishes (NTC-dishes), they were selectively isolated from the other types of cells on the basis of this feature (7, 8).

### Establishment of IPLuM and subculturing of immortalized cells

Lentiviral particles carrying the SV40 large T antigen (SV40LT) gene and the porcine telomerase reverse transcriptase (pTERT) gene were prepared as described previously (9). PLuM were infected with these lentiviral particles in the presence of 6 μg/mL of Polybrene (Nacalai Tesque), and IPLuM were eventually generated.

For the IPLuM subculturing, cells (1 × 10^6^) were seeded in 90-mm NTC-dishes (Sumitomo Bakelite Co., Ltd.) and continuously passaged every 4–5 days. At each passage, the cells were detached using TrypLE express solution (Thermo Fisher Scientific, Waltham, MA), and the number of harvested cells was measured using a Bio-Rad TC20 automated cell counter. Immortalized porcine intestinal macrophages (IPIM) and immortalized porcine kidney-derived macrophages (IPKM), which are highly sensitive to the field ASFV isolates and cell-adapted ASFV isolate reported in our recent studies (6, 10), were also passaged in the same way.

### PCR analysis

The successful transduction of the SV40LT and pTERT genes was confirmed by genomic DNA PCR. A forward primer sequence was designed within a lentiviral vector backbone, and reverse primer sequences were designed within the SV40LT or pTERT gene. The PCR products derived from the SV40LT and pTERT genes were 128 and 143 base pairs (bp) long, respectively. Genomic DNA was extracted from IPIM or IPLuM using NucleoSpin tissue kits (Takara Bio Inc., Shiga, Japan) and added as templates for PCR amplification using KOD FX DNA polymerase (Toyobo Co., Ltd., Osaka, Japan), according to the manufacturer's instructions. The PCR products were analyzed by polyacrylamide gel electrophoresis and visualized *via* GelGreen^TM^ staining (Biotium, Inc., Fremont, CA).

### Immunocytochemistry

IPLuM were seeded in 8-well chamber slides (Asahi Glass Co., Ltd., Tokyo, Japan) at a density of 1.5 × 10^5^ cells/well. After being washed once with DPBS, the cells were fixed using 4% paraformaldehyde phosphate buffer solution (Nacalai Tesque), permeabilized with 1% Triton X-100/PBS solution and blocked with Blocking One Histo (Nacalai Tesque). Then, the cells were incubated with the primary antibodies for 1 h at room temperature, and the EnVision system (DAKO, Hamburg, Germany) was used to visualize antibody-antigen reactions, according to the manufacturer's procedure. Cell nuclei were counterstained with Mayer's hematoxylin solution (FUJIFILM Wako). The stained slides were examined under a microscope (Leica, Bensheim, Germany).

The primary antibodies used for the immunocytochemistry were as follows: mouse monoclonal antibodies against CD52 (Bio-Rad, Hercules, CA), CD163 (Bio-Rad), CD169 (Bio-Rad), CD172a (VMRD, Inc., Pullman, WA), CD203a (Bio-Rad), and major histocompatibility complex class II (MHC-II) (Kingfisher Biotech, Inc., St. Paul, MN) and rabbit polyclonal antibodies against ionized calcium-binding adaptor molecule 1 (Iba1) (FUJIFILM Wako).

### Flow cytometry

IPLuM or IPIM (1 × 10^6^) were cultured in 90-mm NTC-dishes for 3 days, before being treated with or without 1 μg/mL lipopolysaccharide (LPS) for 1 day. Then, the cells were detached using 0.02% ethylenediaminetetraacetic acid solution (Sigma) and re-suspended in DPBS (1 × 10^5^ cells/100 μL) containing mouse monoclonal anti-CD163, anti-CD203a, or anti-MHC-II antibodies. The cells were further labeled with Alexa Fluor 488-conjugated anti-mouse IgG antibodies (Thermo Fisher Scientific), and the number of Alexa Fluor 488-labeled cells and their mean fluorescence intensity (MFI) were analyzed using the BD Accuri^TM^ C6 Plus flow cytometer (BD Biosciences). The fluorescence of 40,000 cells was assessed in each experiment. The MFI data are expressed as mean ± standard error of the mean (SEM) values (*n* = 3), and the mean values were analyzed with one-way analysis of variance followed by Dunnett's post-hoc test using the software GraphPad InStat 3 for Windows. Statistical significance was set at *p* < 0.05.

### Immunoblotting

IPLuM (4×10^5^ cells/well in a 24-well plate) were stimulated with muramyl dipeptide (MDP) (InvivoGen) or LPS (Sigma) in serum-free DMEM at the concentrations indicated. After being incubated at 37°C for 3 days, the culture supernatants were collected, and the cells were lysed with 200 μL ice-cold lysis buffer [50 mM Tris-HCl (pH 7.4), 150 mM NaCl, 0.5% Triton X-100, and 0.5% sodium deoxycholate] containing cOmplete^TM^ mini protease inhibitor (Roche). Equal volumes of culture supernatant and cell lysate (25 μL) were separated by sodium dodecyl sulfate-polyacrylamide gel electrophoresis and electro-blotted onto polyvinylidene difluoride membranes (Millipore). The membranes were incubated with primary antibodies, before being incubated with horseradish peroxidase (HRP)-conjugated secondary antibodies or HRP-conjugated streptavidin. The target proteins were revealed using Chemi-Lumi One ultra (Nacalai Tesque) and detected using a C-DiGit blot scanner (LI-COR, Inc., Lincoln, NE).

The primary antibodies used for the immunoblotting were as follows: biotinylated antibodies against interleukin-1 (IL-1)α (R&D Systems, Minneapolis, MN), IL-1β (R&D Systems), and IL-18 (R&D Systems); rabbit polyclonal antibodies against lactose dehydrogenase (LDH); and mouse monoclonal antibodies against actin.

### Phagocytotic assay using pHrodo-labeled *E. coli* BioParticles

IPLuM (1×10^6^) were cultured in 35-mm glass-bottomed dishes (Asahi Glass Co., Ltd.) containing growth medium. The next day, 20 μg/mL of pHrodo dye-conjugated *E. coli* BioParticles (Thermo Fisher Scientific) were added, and the cells were subjected to time-lapse recording at 37°C for 5 h using an inverted fluorescence microscope (Olympus IX-81, Tokyo, Japan). The mean intensity of the fluorescence emitted by the pHrodo was quantified by analyzing the captured photographs using the software MetaMorph, version 7.6 (Molecular Devices, Downingtown, PA). The data are expressed as mean ± SEM values (*n* = 3).

### ASFV growth assay

The ASFV field isolates Armenia07, Kenya05/Tk-1, and Espana75 were courteously provided by Dr. Sanchez-Vizcaino (Universidad Complutense de Madrid, Spain). These isolates were routinely maintained in PAM cell cultures and stored in aliquots at −80°C until use. The Lisbon60 isolate was kindly provided by Dr. Genovesi (Plum Island Animal Disease Center, USA) and serially passaged in Vero cell cultures to establish the Vero cell-adapted Lisbon60V viruses.

To evaluate ASFV production, IPLuM and IPKM were seeded in T-25 tissue culture flasks (Sumitomo Bakelite Co., Ltd.) and inoculated with ASFV isolates at a multiplicity of infection (MOI) of 0.001. After the cells had been incubated for 1 h at 37°C, the inoculum was removed, the cells were washed three times with DPBS, and then the growth medium was added. The culture supernatants were collected at 1, 2, 3, 4, and 5 days post-inoculation, and the viral titers of the IPKM cell cultures were examined based on cytopathic effects, as described in a previous study (6). Viral titers are expressed as TCID_50_/mL (the 50% tissue culture infectious dose per mL). All experiments with ASFV were performed at the Biosafety Level 3 facility of NIAH and were approved by the Japanese national authority (Permit No. 32).

Statistical analyses were performed using the KaleidaGraph software (Synergy Software, Reading, PA, USA). The Student's *t*-test was used for paired data, and differences associated with *p*-values < 0.05 were considered significant.

## Results

### Characterization of IPLuM

In the mixed culture of porcine primary parenchymal lung cells, PLuM became loosely attached to the cell sheet that formed at the bottom of the T-75 tissue culture flasks. They were collected from the culture supernatant by centrifugation and isolated from the other types of cells based on their ability to adhere to NTC-dishes.

**Figure 1:**
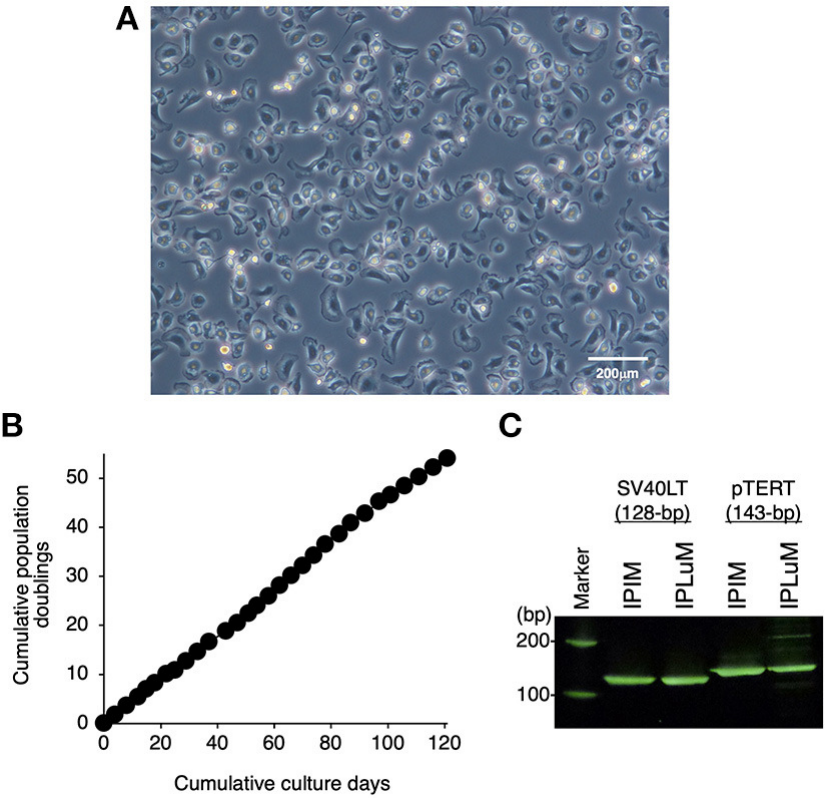
Establishment of the IPLuM cell line. The morphology of IPLuM was examined under a phase-contrast microscope **(A)**. The cumulative population doublings of the IPLuM were plotted against the duration of the culture period (in days) **(B)**. The PCR products generated from the SV40LT (128 bp) and pTERT (143 bp) genes were detected by genomic DNA PCR analysis of IPIM and IPLuM **(C)**.

Then, the PLuM were immortalized by transfecting them with both SV40LT and pTERT genes using lentiviral vectors, and proliferating IPLuM were successfully established. They exhibited a typical macrophage-like morphology with ruffled membranes and cell processes (Figure 1A). They were stably passaged for at least 54 population doublings up to 121 days (Figure 1B). The transduction of the immortalizing genes was confirmed by genomic DNA PCR analysis (Figure 1C).

Immunostaining data showed that the IPLuM were positive for DC/macrophage markers (Iba-1, CD172a, and CD203a; Supplementary Figures 1A,B). Some populations of IPLuM were also clearly positive for markers that are specific to distinct subsets of DC/macrophages (CD163, CD169, and MHC-II) after 3 days of culture (Supplementary Figures 1A,B). Of note, the IPLuM were negative for a monocyte marker, CD52 (Supplementary Figures 1A,B).

### Flow cytometric analysis of CD163, MHC-II, and CD203a expression in IPLuM

**Figure 2:**
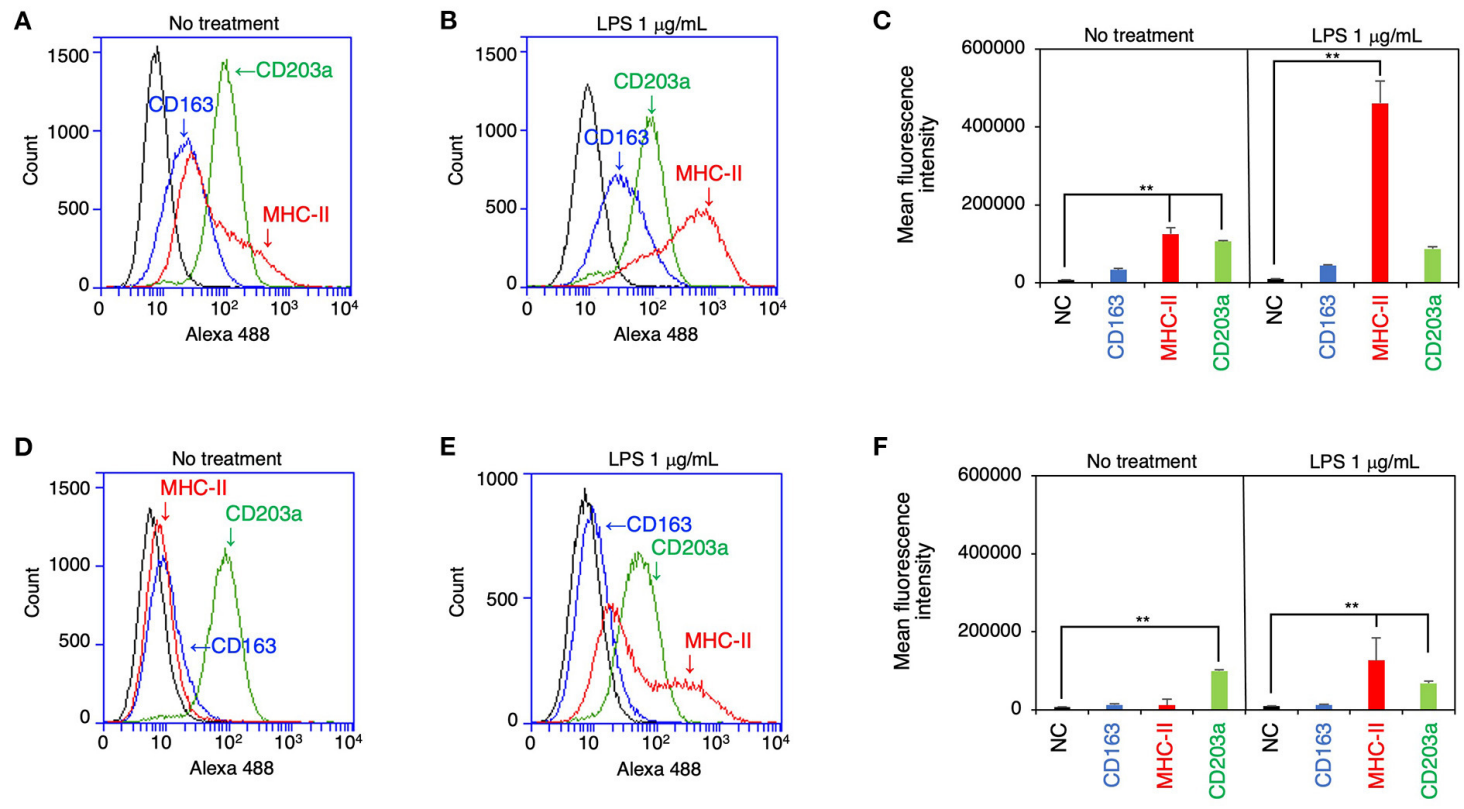
Flow cytometric analyses of IPLuM and IPIM. IPLuM **(A–C)** or IPIM **(D–F)** were treated with **(B,E)** or without 1 μg/mL LPS **(A,D)** for 1 day. Then, the cells were reacted with mouse monoclonal anti-CD163 (blue line), anti-CD203a (green line), or anti-MHC-II (red line) antibodies, before being labeled with Alexa Fluor 488-conjugated anti-mouse IgG antibodies (Alexa 488). The cells treated with Alexa 488 alone were used as a negative control (NC) (black line). The flow cytometry histograms are representative of three independent experiments **(A,B,D,E)**, and the MFI data are expressed as mean ± SEM values (*n* = 3, **p < 0.01 vs. NC) **(C,F)**.

The expression of CD163, MHC-II, and CD203a by IPLuM was quantitatively analyzed by flow cytometry and compared with that seen on IPIM. The IPLuM exhibited higher frequencies of CD163-positive and MHC-II-positive cells than the IPIM (Figures 2A,D). The MFI value of the MHC-II-positive cells was significantly higher than that of the unstained cells among the IPLuM (Figure 2C), but not among the IPIM, in the absence of LPS (Figure 2F). CD203a was constitutively expressed by both the IPLuM and IPIM (Figures 2A,C,D,F).

In the presence of LPS, both cell lines showed a marked increase in the frequency of MHC-II-positive cells, whereas the frequency of CD203a-positive cells was slightly reduced among both the IPLuM and IPIM (Figures 2B,C,E,F). The frequency of CD163-positive cells among IPLuM or IPIM was not affected by LPS treatment (Figures 2B,C,E,F).

### Inflammatory responses and phagocytotic activity of IPLuM

**Figure 3:**
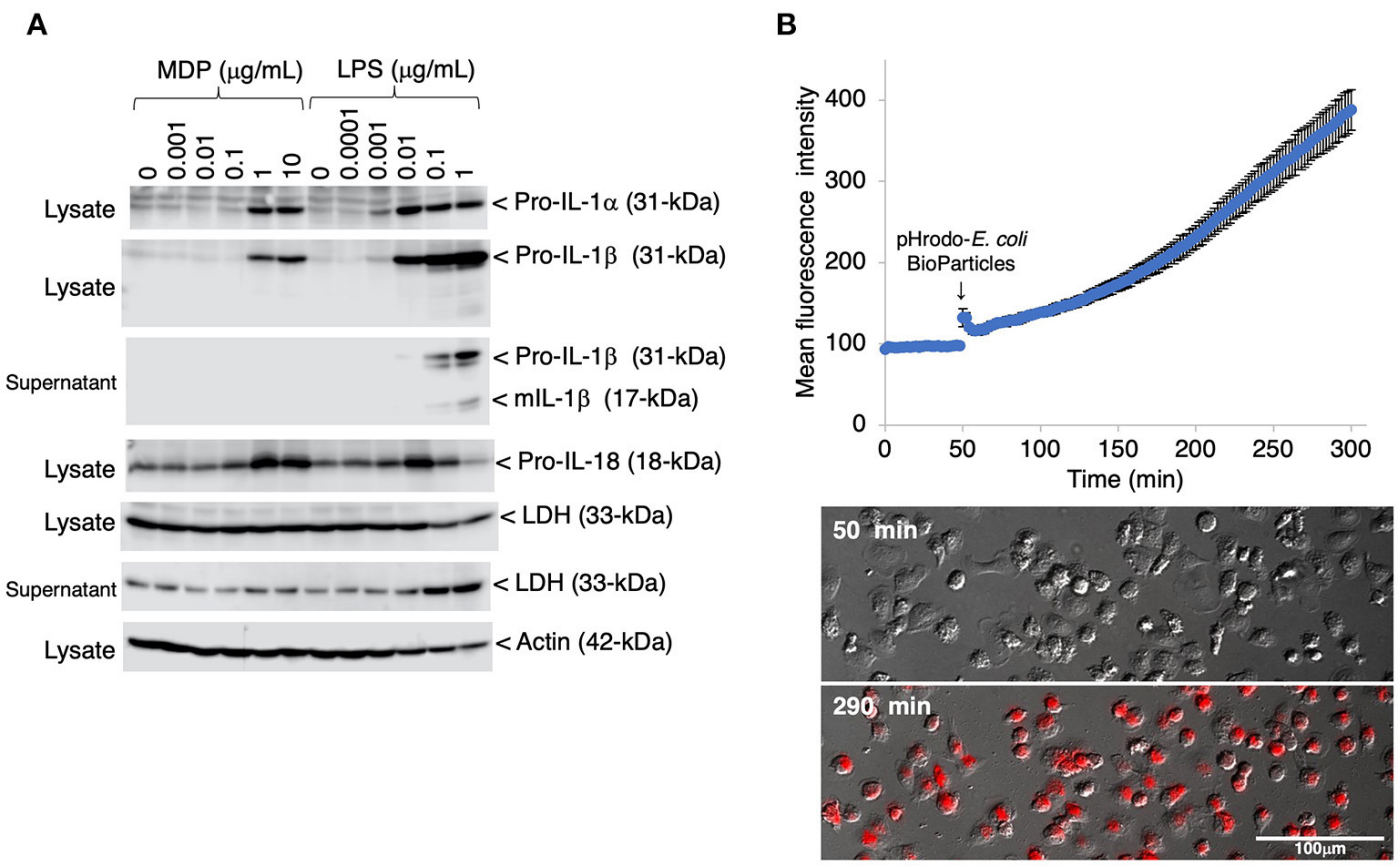
Inflammatory responses and phagocytotic activity of IPLuM. The dose-dependent production of pro-IL-1α and pro-IL-1β was detected in cell lysates from IPLuM that had been stimulated with MDP or LPS for 3 days [**(A)***, first and second panels*]. The secretion of mIL-1β from the LPS-treated IPLuM into the culture supernatant was also detected [**(A)***, third panel*]. The production of pro-IL-18 was detected after MDP/LPS stimulation [**(A)**, fourth panel]. Dose-dependent LDH leakage from the LPS-treated IPLuM was observed [**(A)***, fifth and sixth panels*]. Actin was used as an internal control [**(A)***, seventh panel*]. The blots are representative of three independent experiments. IPLuM were treated with pHrodo-labeled *E. coli* BioParticles and monitored *via* time-lapse fluorescence imaging of live cells **(B)**. The mean intensity of the fluorescence emitted by pHrodo was plotted against the duration of the culture period (in minutes) **(B)**. The data are expressed as mean ± SEM values (*n* = 3). pHrodo-derived fluorescence was observed in almost all of the IPLuM [red in lowest panel of **(B)**].

To evaluate the inflammatory responses of the IPLuM, the effects of bacterial cell wall components, MDP and LPS, were investigated in IPLuM. These stimuli elicited the production of the precursor forms of IL-1α (pro-IL-1α) and IL-1β (pro-IL-1β), which are known to be potent pro-inflammatory cytokines, in a dose-dependent manner (Figure 3A, first and second panels). In addition, LPS-induced secretion of the mature active form of IL-1β (mIL-1β) into the culture supernatant was detected (Figure 3A, third panel). Dose-dependent production of the precursor form of IL-18 (pro-IL-18), another pro-inflammatory cytokine belonging to the IL-1 family, was detected in MDP-treated IPLuM, while LPS-induced pro-IL-18 production peaked in the presence of 0.01 μg/mL LPS and decreased at higher LPS concentrations (Figure 3A, fourth panel). Higher concentrations of LPS also elicited LDH release into the culture supernatant, whereas MDP treatment did not affect its release (Figure 3A, sixth panel).

To evaluate the phagocytotic activity of the IPLuM, IPLuM that had been treated with pHrodo-labeled *E. coli* BioParticles were monitored by time-lapse fluorescence imaging of live cells. The mean intensity of pHrodo-derived fluorescence increased in a time-dependent manner, and almost all cells exhibited such fluorescence, which represented phagosomal maturation, after 4 h incubation (Figure 3B).

### Propagation of ASFV isolates in IPLuM

**Figure 4:**
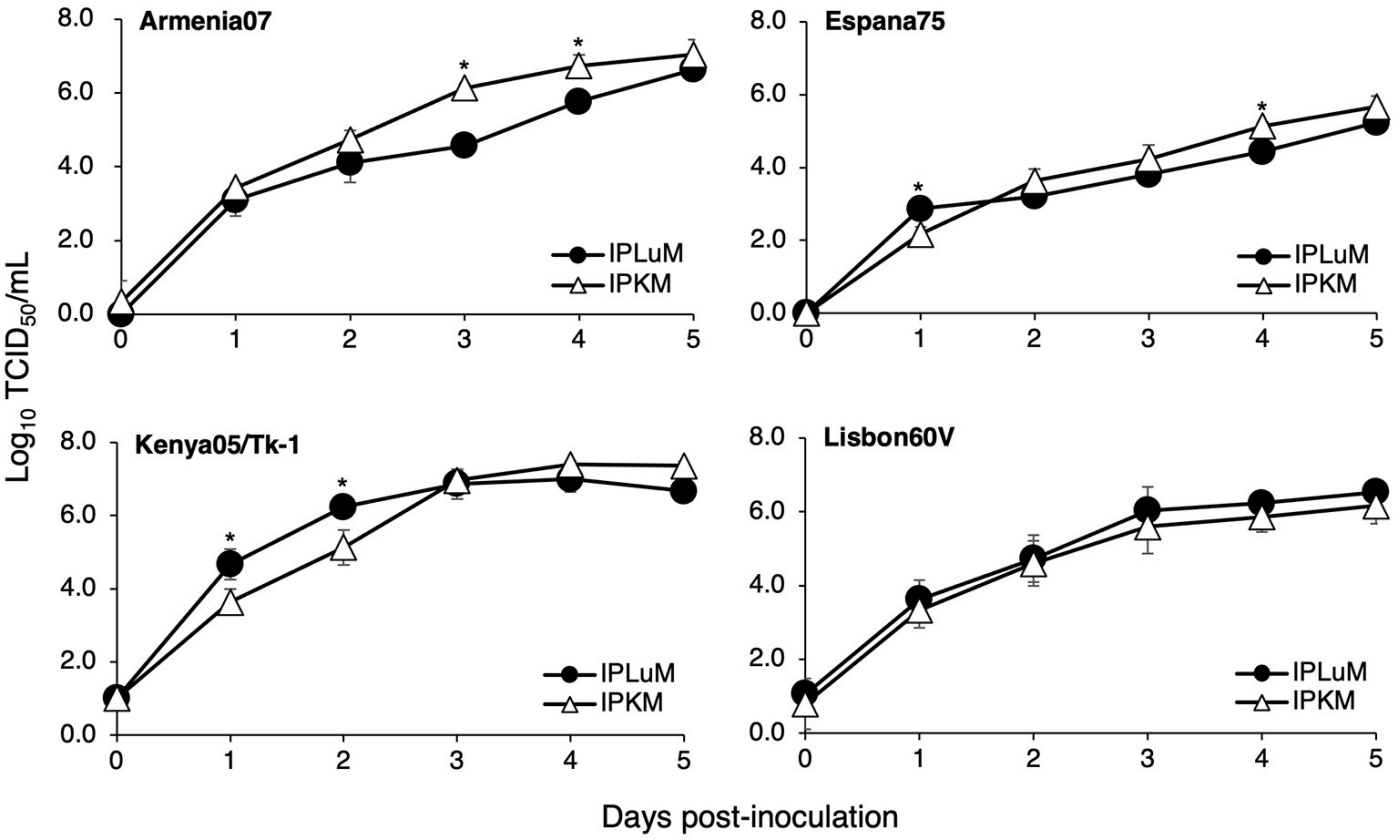
Comparison of ASFV production in IPLuM and IPKM. Cell cultures were infected with Armina07, Kenya05/Tk-1, Espana75, or Lisbon60V isolates (MOI = 0.001). Culture supernatant samples were collected at the indicated timepoints. Viral production in the IPLuM **(closed circles)** and IPKM **(open triangles)** cell cultures was estimated by titration experiments with the IPKM. Data represent the mean and standard deviation of three experiments. Asterisks indicate statistically significant differences in viral production between the IPLuM and IPKM cell cultures (*p < 0.05).

Finally, we examined whether IPLuM are susceptible to ASFV infection and support the intracellular replication of the virus. As shown in Figure 4, various ASFV strains, Armenia07, Kenya05/Tk-1, Espana75, and Lisbon60V, propagated in IPLuM cell cultures as efficiently as in IPKM cell cultures.

## Discussion

Several immortalized PAM (IPAM) cell lines have been established, and their utility for *in vitro* cultures of viral pathogens has been examined (11, 12). In the present study, we demonstrated that PLuM preparations contain not only PAM, but also other types of MNP of porcine lung origin, including pulmonary intravascular macrophages and interstitial macrophages (5, 13), and that IPLuM are phenotypically different from IPAM. In particular, pulmonary intravascular macrophages are abundant in the lungs of pigs and have been reported to support the growth of PRRSV at high titers (5). They have also been reported to be preferential target cells for ASFV infection (14, 15). Further characterization of IPLuM will make it possible to develop unique *in vitro* models for studying host-pathogen interactions in porcine respiratory tissues.

CD163 is mainly expressed in macrophages and is used as a phenotypic marker of anti-inflammatory M2 subtypes (16). This notion is supported by the previous finding that CD163-positive porcine parenchymal lung cells predominantly exhibited macrophage phenotypes (4). Furthermore, PAM were reported to be CD163, MHC-II, and CD172a triple-positive cells (4). Although it is considered that IPLuM contain PAM subpopulations, multicolor immunofluorescence analysis will be required to confirm this.

As for other cell surface markers, CD52 is expected to be expressed at much higher levels on monocytes than on mature macrophages (17). IPLuM were shown to be negative for CD52 (Supplementary Figure 1), indicating that they are not of monocytic cell origin. In contrast, almost all of the IPLuM were positive for CD172a, which is expressed on cells of myeloid origin and is indicative of a DC and macrophage-like phenotype (18). In addition, higher expression of MHC-II, a well-known marker of mature DC (19), was detected in some populations of IPLuM. In this context, a previous extensive study demonstrated that MHC-II-positive, CD172a-positive, and CD163-low/intermediate-expressing porcine parenchymal lung cells exhibit monocyte-derived DC/macrophage phenotypes (4). Considering that CD163-low/intermediate-expressing cells were mainly found among IPLuM, it is likely that IPLuM include monocyte-derived DC/macrophage subpopulations.

Treatment with MDP and LPS increased the expression of the precursor forms of IL-1α, IL-1β, and IL-18, suggesting that it induced pro-inflammatory reactions by IPLuM. The LPS-induced secretion of the mature active form of IL-1β represents the expression of the functional porcine inflammasome system in IPLuM (20). Conversely, we noticed that IL-18β expression was reduced in IPLuM that had been stimulated with higher concentrations of LPS. LPS-induced cell damage accompanied by LDH release was also observed in the higher concentration range. It is speculated that cell damage may be linked to the reduced expression of IL-18 seen in LPS-stimulated IPLuM.

ASFV is a highly pathogenic virus with a marked tropism for cells of the monocyte-macrophage lineage (21). Similar to the IPKM and IPIM cell lines reported in our recent studies (6, 10), IPLuM were confirmed to be susceptible to ASFV infection and to facilitate the propagation of the virus very efficiently. It has been reported that the titers of replicating ASFV isolates were much lower in IPAM than in primary PAM (22). Thus, this may suggest that the induction of immortalization alters the character of macrophages and reduces their susceptibility to ASFV. However, the immortalization protocols we established in a previous study (9) have minimal effects on macrophage functions, and the resultant cells remain susceptible to ASFV infection.

In conclusion, we produced a novel porcine lung-derived MNP cell line, IPLuM, which exhibited DC/macrophage-like phenotypes rather than monocyte phenotypes. This cell line is a useful model that reflects the porcine lung microenvironment *in vitro*. Furthermore, it may be useful for investigating the host-pathogen interactions that occur in porcine respiratory diseases.

## Data availability statement

The original contributions presented in the study are included in the article/Supplementary material, further inquiries can be directed to the corresponding authors.

## Ethics statement

The animal study was reviewed and approved by the animal care committee of the Institute of Agrobiological Sciences and the National Institute of Animal Health, National Agriculture and Food Research Organization. Written informed consent was obtained from the owners for the participation of their animals in this study.

## Author contributions

TT and KM conceived, designed the experiments, and analyzed the data. TT, KM, and KH performed the experiments. TT, KM, SS, SH, TK, and HU contributed reagents/materials/analytical tools. TT, KM, and TK wrote the manuscript. All authors contributed to the article and approved the submitted version.

## Funding

This study was conducted as part of the research project on Regulatory research projects for food safety, animal health and plant protection (JPJ008617. 20319736) funded by the Ministry of Agriculture, Forestry and Fisheries of Japan.

## Conflict of interest

The authors declare that the research was conducted in the absence of any commercial or financial relationships that could be construed as a potential conflict of interest.

## Publisher's note

All claims expressed in this article are solely those of the authors and do not necessarily represent those of their affiliated organizations, or those of the publisher, the editors and the reviewers. Any product that may be evaluated in this article, or claim that may be made by its manufacturer, is not guaranteed or endorsed by the publisher.

## Supplementary material

Supplementary Figure 1 Immunocytochemical characterization of IPLuM. The IPLuM were seeded in 8-well chamber slides and cultured for 1 day **(A)** or 3 days **(B)**. Then, the cells were fixed using 4% paraformaldehyde phosphate buffer solution and immunostained with specific antibodies against cell markers of MNP (brown). No specific staining was observed when the cells were treated without primary antibodies [NC, negative control in (**A,B)**]. All nuclei were counterstained with hematoxylin (blue). Images are representative of three independent experiments.
