# Supplementary figures and images for: Establishment and characterization of the immortalized porcine lung-derived mononuclear phagocyte cell line
Source: Front Vet Sci. 2022 Nov 18;9:1058124. doi: 10.3389/fvets.2022.1058124 (PMC9715978; doi:10.3389/fvets.2022.1058124)

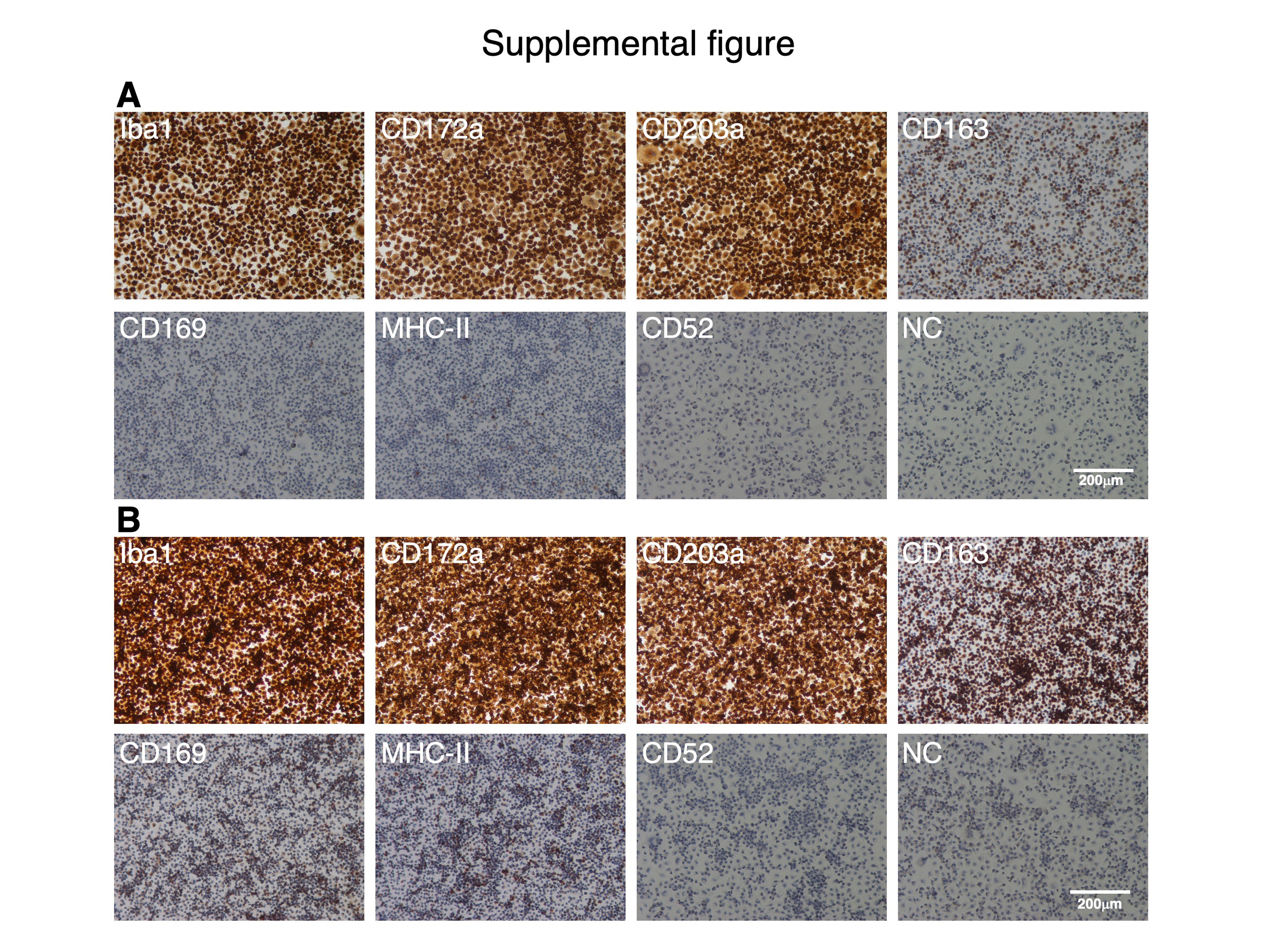

Supplement: Supplementary Figure 1 — Immunocytochemical characterization of IPLuM. The IPLuM were seeded in 8-well chamber slides and cultured for 1 day (A) or 3 days (B). Then, the cells were fixed using 4% paraformaldehyde phosphate buffer solution and immunostained with specific antibodies against cell markers of MNP (brown). No specific staining was observed when the cells were treated without primary antibodies [NC, negative control in (A,B)]. All nuclei were counterstained with hematoxylin (blue). Images are representative of three independent experiments. [file Image_1.JPEG]
